# An Earthworm Riddle: Systematics and Phylogeography of the Spanish Lumbricid *Postandrilus*

**DOI:** 10.1371/journal.pone.0028153

**Published:** 2011-11-29

**Authors:** Marcos Pérez-Losada, Jesse W. Breinholt, Pablo G. Porto, Manuel Aira, Jorge Domínguez

**Affiliations:** 1 Centro de Investigação em Biodiversidade e Recursos Genéticos (CIBIO), Universidade do Porto, Campus Agrário de Vairão, Vairão, Portugal; 2 Department of Biology, Brigham Young University, Provo, Utah, United States of America; 3 Departamento de Ecoloxía e Bioloxía Animal, Universidade de Vigo, Vigo, Spain; American Museum of Natural History, United States of America

## Abstract

**Background:**

As currently defined, the genus *Postandrilus* Qui and Bouché, 1998, (Lumbricidae) includes six earthworm species, five occurring in Majorca (Baleares Islands, western Mediterranean) and another in Galicia (NW Spain). This disjunct and restricted distribution raises some interesting phylogeographic questions: (1) Is *Postandrilus* distribution the result of the separation of the Baleares-Kabylies (BK) microplate from the proto-Iberian Peninsula in the Late Oligocene (30–28 Mya) – vicariant hypothesis? (2) Did *Postandrilus* diversify in Spain and then colonize the Baleares during the Messinian salinity crisis (MSC) 5.96–5.33 Mya – dispersal hypothesis? (3) Is the distribution the result of a two-step process – vicariance with subsequent dispersal?

**Methodology/Principal Findings:**

To answer these questions and assess *Postandrilus* evolutionary relationships and systematics, we collected all of the six *Postandrilus* species (46 specimens – 16 locations) and used *Aporrectodea morenoe* and three *Prosellodrilus* and two *Cataladrilus* species as the outgroup. Regions of the nuclear 28S rDNA and mitochondrial 16S rDNA, 12S rDNA, ND1, COII and tRNA genes (4,666 bp) were sequenced and analyzed using maximum likelihood and Bayesian methods of phylogenetic and divergence time estimation. The resulting trees revealed six new *Postandrilus* species in Majorca that clustered with the other five species already described. This Majorcan clade was sister to an Iberian clade including *A. morenoe* (outgroup) and *Postandrilus bertae*. Our phylogeny and divergence time estimates indicated that the split between the Iberian and Majorcan *Postandrilus* clades took place 30.1 Mya, in concordance with the break of the BK microplate from the proto-Iberian Peninsula, and that the present Majorcan clade diversified 5.7 Mya, during the MSC.

**Conclusions:**

*Postandrilus* is highly diverse including multiple cryptic species in Majorca. The genus is not monophyletic and invalid as currently defined. *Postandrilus* is of vicariant origin and its radiation began in the Late Oligocene.

## Introduction

While attending a scientific meeting in 1997, Marcel B. Bouché collected earthworms in eight locations in the Island of Majorca (Baleares, western Mediterranean). The study of that material led to the discovery of five new earthworm species that were included in a new genus named *Postandrilus* [1]. The authors also included *Postandrilus bertae*, formerly *Cernosvitovia bertae* – an earthworm species from Galicia (NW Spain) that, hesitantly, Díaz-Cosín et al. [2] had previously assigned to the eastern European genus *Cernosvitovia*. According to Qiu and Bouché [1], *Postandrilus* (Oligochaeta, Lumbricidae) can be differentiated from other lumbricids by its male genitalia. *Postandrilus* has their male pores located at the beginning of the *tubercula pubertatis*, far from segment 15 (the common position in the family Lumbricidae), and presents a larger and narrower crop and gizzard, supposedly to facilitate sperm transfer throughout the sperm ducts. *Postandrilus* earthworms are of medium to large size (100–420 mm), sedentary (i.e., expected low dispersal ability) and considered endogeic because all lack pigmentation, live in galleries (sometimes deeper than 20 cm) and their casts are comprised exclusively of mineral soil (pers. obs.).

Qiu and Bouché [1] defined three *Postandrilus* subgenera and six species based on the male reproductive system: (1) *P. Galiciandrilus*, which only includes the Iberian *P. Galiciandrilus bertae*; (2) *P. Merandrilus*, whose type species is *P. Merandrilus majorcanus* and includes also *P. Merandrilus medoakus*, both from Majorca; and (3) *P. Postandrilus*, whose type species is *P. Postandrilus palmensis* and includes also *P. Postandrilus lavellei* and *P. Postandrilus sapkarevi*, also from Majorca. The subgenus *P. Postandrilus* differs from the other two subgenera by having two pairs of seminal vesicles instead of one, while *P. Galiciandrilus*, the most distinct of the three subgenera, has 12 spermathecae located in segments 15–20 instead of four at segments 10 and 11. Furthermore *P. Merandrilus* lacks epididymus and seminal capsules and are the largest earthworms from this group sizing up to 420 mm, whereas *P. Postandrilus* and *P. Galiciandrilus* are medium sized (shorter than 250 mm) with the exception of *P. Postandrilus sapkarevi* that may reach up to 350 mm. Despite these morphological differences, the taxonomic status, evolutionary relationships and diversity of *Postandrilus* and its subgenera and species have not been assessed using a phylogenetic approach.

**Figure 1:**
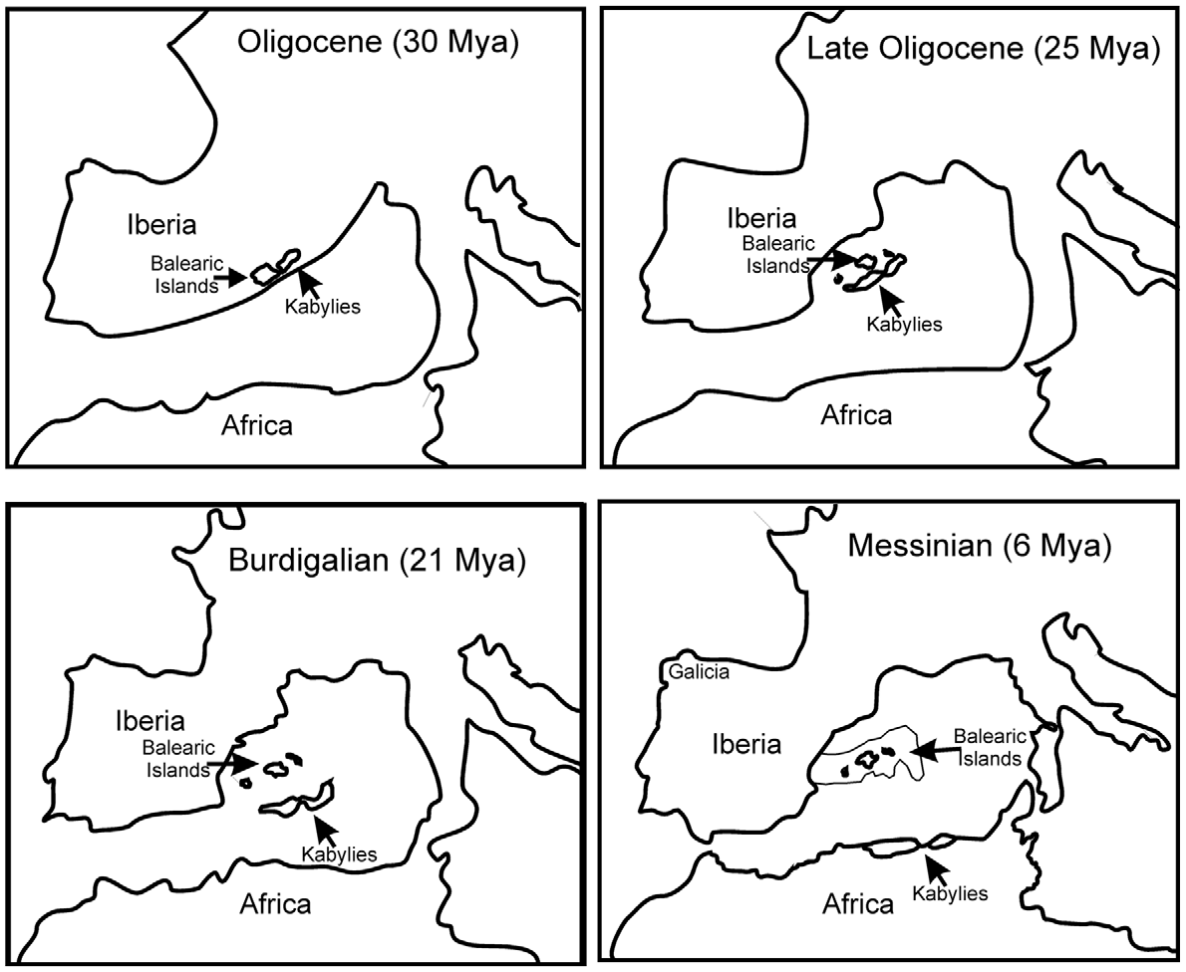
Paleomaps of the developing western Mediterranean from the Eocene to the Pleistocene (modified from [**4]**, [55****]). In the Messinian map, the grey area indicates the position of the land bridge connecting the Balearic Islands to the Iberian Peninsula.

As indicated above, five of the six *Postandrilus* are endemic to the Island of Majorca, the largest of the three main islands of the Baleares archipelago, and the other species has only been found in a small area of Galicia (NW Spain, Fig. 1). Such a restricted and disjunct geographical distribution is puzzling, considering the geographical distance separating both regions and the geological history of the Iberian Peninsula. Before the Oligocene, the Baleares together with the Kabylies (Algeria), Corsica, Sardinia, the Tuscan archipelago and the Calabro-Peloritan massif (Italy) and the internal parts of the Betic-Rif cordillera (Spain and Morocco, respectively) were part of the Hercynian belt, a Paleozoic mountain chain situated in Iberia and southern Europe [3], [4], [5]. In the Early Oligocene this Hercynian massif was fragmented into microplates that dispersed throughout the western Mediterranean. According to tectonic reconstructions [3], [4], in the Late Oligocene (30–28 million years ago – Mya) the Baleares-Kabylies (BK) microplate and the Corso-Sardinian-Calabro-Peloritan (CSCP) microplate drifted off the proto-Iberian Peninsula (Fig. 1). Around 25 Mya, the BK microplate began to rotate clockwise until the Balearic Islands reached their current position (∼21 Mya) and separated from the Kabylies terrane, which continued to drift south toward North Africa. The CSCP microplate drifted eastward until about 5 Mya split into two smaller microplates (Corso-Sardinian and Calabro-Peloritan), which continued to move until they reached their current positions. From this point on we will only focus on the Balearic Islands. For most of the Miocene and until the Messinian salinity crisis (MSC, 5.96–5.33 Mya), an event characterized by dramatic drying and salinity increase of the Mediterranean Sea due to isolation from the Atlantic Ocean [6], [7], [8], the Baleares remained isolated from the continent. During the MSC the western Mediterranean water level dropped >1,000 m, allowing the reconnection of the Baleares to eastern Spain for a short period of time [9]. Subsequent Pleistocene (1.8–0.01 Mya) glaciations also caused severe drops in sea-level (up to 150 m), which allowed episodic contacts among Balearic islands but not between the islands and mainland [10], [11].

Considering the current distribution of the *Postandrilus* species and the geological history of the Baleares, we asked the following phylogeographic questions: (1) Was the radiation of *Postandrilus* concomitant with the fragmentation of the Hercynian belt and the separation of the BK microplate (vicariant hypothesis)? (2) Did *Postandrilus* evolve in situ in proto-Iberia and then actively or passively (e.g., birds, ruminants; [12]) migrated to Majorca during the MSC (dispersal hypothesis)? (3) Since both hypotheses are non-exclusive, is *Postandrilus* distribution the result of vicariance and subsequent dispersal? To solve this riddle we adopted an integrative approach, including molecular phylogenetics and divergence time estimation analyses in the context of the geological history of the Iberian Peninsula and the Baleares Islands.

## Results

**Figure 2:**
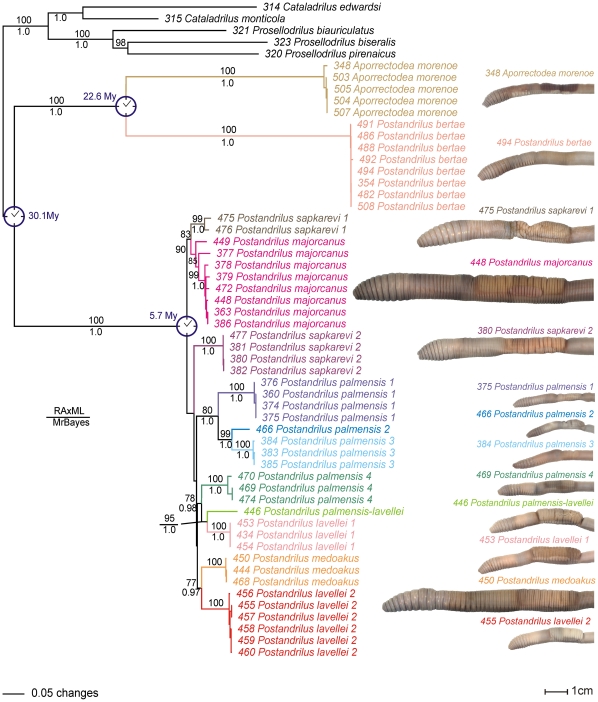
Maximum likelihood tree. Branch lengths are shown proportional to the amount of change along the branches. Bootstrap proportions (if≥70%) and Bayesian posterior probabilities (if≥0.95) are shown above and below the branches, respectively. Specimen photographs for each lineage are also shown. Only the numerical part of the specimen codes is shown for simplicity.

Our maximum likelihood (ML) and Bayesian phylogenetic analyses generated similar topologies and clade support (Fig. 2). Most of the internal nodes were well supported, but three of the nodes involving the Majorcan species were not, despite the high number of characters (4,666 aligned sites) and sophisticated phylogenetic methods used. *Postandrilus*, as stated in Qiu and Bouché [1], did not form a monophyletic assemblage; instead, all of our trees revealed a strongly supported (bp = 100 and p*P* = 1.0) Iberian clade clustering *P. bertae* (one lineage) and the outgroup species *A. morenoe* (one lineage) and a strongly supported (bp = 100% and p*P* = 1.0) Majorcan clade including all the other *Postandrilus*. A monophyletic Iberian-Majorcan *Postandrilus* clade was rejected by the S–H test (*P*<0.001) and presented a p*P*<0.001. Tree branch lengths among *P. bertae* and the Majorcan *Postandrilus* clade ranged from 1.16 to 1.30. However, branch lengths among congeneric and conspecific Majorcan *Postandrilus* taxa ranged from 0.08 to 0.17 and from 0 to 0.06, respectively. This highlights the genetic differences between peninsular and insular *Postandrilus.* Congeneric branch lengths were higher (0.13 to 0.17) when we excluded the 475 and 476 *P. sapkarevi* specimens. These two specimens failed to amplify a large section of the rRNA region (∼1 Kb), so the branch connecting them to *P. majorcanus* was relatively short compared to the other branches in the Majorcan clade (Fig. 2). Congeneric and conspecific branch lengths reported here among Majorcan *Postandrilus* were similar to those described for other earthworms falling in the same taxonomic rankings. Branch lengths between *P. bertae* and the Majorcan *Postandrilus* were proper of confamilial taxa [13], [14], [15], [16], [17].

The Majorcan *Postandrilus* clade included four *P. palmensis* lineages, two *P. sapkarevi*, two *P. lavellei* and one of each *P. majorcanus*, *P. medoakus* and *P. palmensis/lavellei* (Fig. 2). All these lineages were supported by high bp (≥70) or p*P* (≥0.95). *Postandrilus palmensis-lavellei* presented morphological features common to both species (e.g., the location and length of the clitellum and *tubercula pubertatis*) and also fell in between them in the trees. *Postandrilus palmensis*, *P. sapkarevi* and *P. lavellei* did not form monophyletic assemblages. The alternative monophyletic hypotheses were rejected by the S–H test (*P*<0.001) and presented p*P*<0.001. Within the main *P. palmensis* clade (lineages 1 to 3), branch lengths among these three subclades were similar to those observed among other valid *Postandrilus* species (e.g., *P. sapkarevi* 1– *P. majorcanus* or *P. lavellei* 2 – *P. medoakus* clades) and fell in the range of those previously described in congeneric earthworms [13], [14], [15], [16], [17].

Hence, our phylogenetic analyses revealed a total of 11 species in Majorca, five potentially corresponding to those species identified by Qiu and Bouché [1] since we collected them in the same localities (except for *P. medoakus*), and another six (two also from Qiu and Bouché [1] type localities) that could potentially correspond to new *Postandrilus* species. Of the 11 lineages, four of these taxa were collected in the same location (*P. sapkarevi 2* – *P. palmensis 3* and *P. palmensis 4* – *P. medoakus*), but the other seven were not. Interestingly, not all the samples collected in different locations formed different lineages, thus *A. morenoe* (2 locations), *P. bertae* (2), *P. majorcanus* (5), and *P. palmensis 1* (2) showed shallow (conspecific) genetic differences.

Our phylogenetic trees suggested a single radiation of *Postandrilus* in the Baleares (Fig. 2). The divergence time estimation analysis in BEAST indicated that the split between the Iberian *P. bertae* and *A. morenoe* and the Majorcan *Postandrilus* lineages took place 30.1 (22.2–38.7) Mya. Similarly, the Majorcan *Postandrilus* would have diversified 5.7 (4.3–7.3) Mya and the Iberian *P. bertae* would have diverged from *A. morenoe* 22.6 (16.8–29.3) Mya. The two former molecular time estimates overlap (i.e., are not significantly different) with the geological ages estimated for the separation of the Baleares-Kabylies microplate from the proto-Iberian Peninsula (30–28 Mya) and the duration of the Messinian salinity crisis (5.96–5.33 Mya), respectively (Fig 1). Our substitution rate estimates [mean (SD) %] for all the partitions were as follows: coding mtDNA = 1.82 (0.3) s/My^−1^, rRNA = 1.12 (0.18) s/My^−1^, tRNA = 0.9 (0.13) s/My^−1^, and 28S = 0.063 (0.011) s/My^−1^. These rates fell within the 95% confidence intervals of the rate priors estimated by us using sequences in Chang et al. [18] and were similar to those reported in other invertebrates including annelids [19], [20]. Interestingly, estimates reported by Novo et al. [17] for their Hormogastridae mitochondrial genes [0.52 (0.2) s/My^−1^)] were lower than those reported here.

## Discussion

### Systematics of *Postandrilus*

Our phylogenetic analyses of the genus *Postandrilus* showed that all the Majorcan taxa formed a relatively uniform and well supported clade that clustered with another very genetically distinct (as indicated by the tree branch lengths) and also well supported clade including two *A. morenoe* (initially part of the outgroup) and two *P. bertae* populations. This result hence suggests that the genus *Postandrilus* is not monophyletic and invalid as currently stated in Qiu and Bouché [1] and Blakemore [21]. Morphologically, *A. morenoe* is relatively different from *Postandrilus*, but also very different from other *Aporrectodea* taxa [22]. *A. morenoe* lacks spermathecae, as other *Aporrectodea* and Lumbricidae has the male pore in segment 15 and presents four pairs of seminal vesicles (segments 9–12). Moreover, it has bilobulate nephridial vesicles instead of the J- or U-shaped ones typical of *Aporrectodea*, and lacks calciferous sacks in segment 10. However, the length, location and appearance of the clitellum and the *tubercula pubertatis* resemble those of *Postandrilus*. Díaz-Cosín et al. [22] also were not certain about the taxonomic status of *A. morenoe* when they described it: “It is difficult to assign this species [*A. morenoe*] to any concrete genus. In Omodeo's (1956) classification it most resembles *Eophila* on account of its size, the number of segments and the clitellum. The lack of calciferous sacs in segment 10 might place it in Gates' *Helodrilus* or *Eisenoides* (Gates 1978). Michaelsen's (1900) classification might place it in *Helodrilus* (*Allolobophora*), Pop's (1948) in *Allolobophora*, and Vedovini's (1973) in *Eophila* or *Helodrilus*”. Other phylogenetic analyses of the Lumbricidae genera performed by our group (unpublished data) also showed that *A. morenoe* is genetically very different from other *Aporrectodea* species and is sister related to *P. bertae*, which confirms its taxonomic uncertainty.

Similarly, *Cernosvitovia bertae* was tentatively included in this genus [2], although as the authors commented “*Cernosvitovia bertae* is easily differentiated from the other species in the genus by several characters. The clitellum is longer, reaching segment 51, while in the other species it goes as far as segment 34 [translated from Spanish]”. In other phylogenetic analyses of the Lumbricidae genera performed by our group (unpublished data), *P. bertae* did not cluster with other *Cernosvitovia* species either. Qiu and Bouché [1], however, included this species in the *Postandrilus* genus based on its male genitalia, but they also highlighted the morphological differences separating *P. bertae* from its insular relatives – mainly the number and position of the spermathecae (six pairs in segments 15 to 20 instead of two pairs in segments 10 and 11).

Therefore, based on all of the previous evidence, we propose to use the taxonomic name *Postandrilus* only for the species of this genus occurring in Majorca. *Postandrilus bertae* should be moved to a new genus different from *Cernosvitovia* [2], although the designation of such genus is beyond the scope of this study. As for the other *Postandrilus* subgenera, our phylogenetic analyses did not support the taxonomic status of *P. Merandrilus* and *P. Postandrilus* as currently stated in Qiu and Bouché [1] and Blakemore [21], since both subgenera did not form reciprocally monophyletic assemblages (Fig. 2).

**Figure 3:**
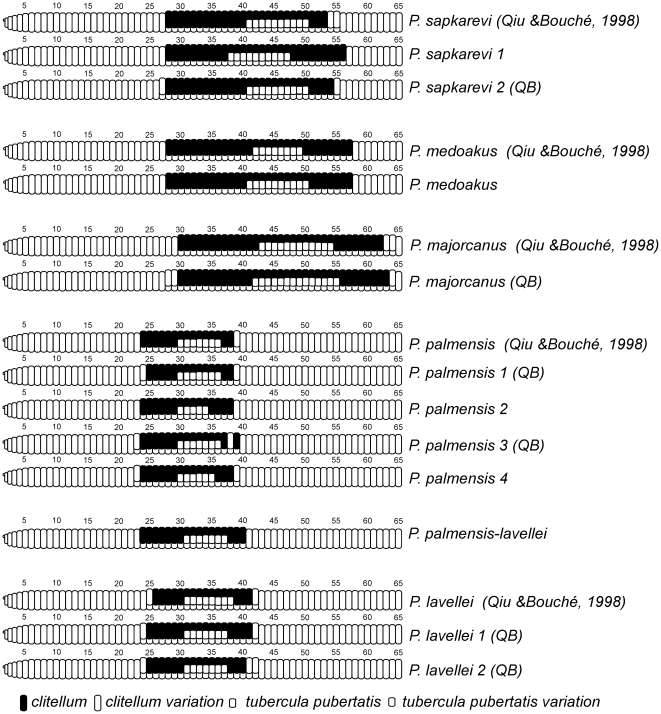
Drawings of the clitellum and *tubercula pubertatis* for the eleven lineages composing the Majorcan *Postandrilus* clade (based on original descriptions by Qiu and Bouché [**1**]). Earthworm lineages sampled in the type localities in Qiu and Bouché [1] are indicated by QB.

Finally, our phylogenetic analyses also revealed six potentially new *Postandrilus* species in Majorca (assuming our *P. medoakus* is the same species sampled by Qiu and Bouché [1]) rendering 11 species, which is more than twice the total number of insular species (five) currently included in the genus. Clade support among these 11 lineages varied from weak (bp<70 and p*P*<0.95) to strong, however all of them showed deep phylogenetic structuring, which is indicative of high (ancient) genetic divergence. Pairs of valid and morphologically identified species collected in the same type localities sampled by Qiu and Bouché [1], presented levels of genetic divergence similar to those observed among pairs of new lineages identified here using molecular phylogenetic analysis. Moreover, no evidence of gene flow was observed between those putative species despite the fact that some of them (*P. palmensis 4* and *P. medoakus*) occur in sympatry, which again validates their taxonomic status. Additionally, these phylogenetic results were also supported by morphological, ecological and genomic evidence. Summarizing, *P. medoakus* was found in a different locality than that sampled in Qiu and Bouché [1], but they presented the same clitellum. However, the *tubercula pubertatis* extend one segment before and after those described by the authors (Fig. 3). This variation in length on the *tubercula pubertatis* may be indicative of a new species, but since Qiu and Bouché [1] only used one adult and two immature specimens for their description, the species variation for this character was not reported. Earthworms of the lineages *P. lavellei* 1 and 2 did not exactly match the clitellum and *tubercula pubertatis* defined by Qiu and Bouché [1] for this species (Fig. 3), although their characteristics fell within the variation reported by the authors. The only two characters we found that separate *P. lavellei* 1 and 2 are the colour of the clitellum after fixation (see Fig. 2) and the length of the *tubercula pubertatis* (Fig. 3). The same rationale described before for the clitellum and *tubercula pubertatis* in *P. lavellei* can be applied to the four *P. palmensis* lineages, except for lineage *P. palmensis* 2, which showed a *tubercula pubertatis* shorter than that observed in the other three *P. palmensis* lineages (Fig. 3). *Postandrilus palmensis-lavellei* presented a clitellum similar to that in *P. palmensis* but its *tubercula pubertatis* resembled that in *P. lavellei* (Fig. 3). Earthworms of the lineage *P. sapkarevi* 2 were collected in the same locality as those in Qiu and Bouché [1] and shared the same morphological characteristics (Fig. 3). On the other hand, earthworms of the lineage *P. sapkarevi* 1 showed a slightly longer clitellum and its *tubercula pubertatis* had the same length but was placed two segments before than in *P. sapkarevi* 2 (Fig. 3). All *P. majorcanus* specimens formed one lineage and, despite their morphological variability, resembled those described by Qiu and Bouché [1] (Fig. 3).

Hence, based on the integrative approach of species delimitation [23], [24], [25], and considering our sampling design, we confirm the taxonomic validity of *P. majorcanus*, *P. medoakus*, *P. palmensis*, *P. lavellei* and *P. sapkarevi*, as described in Qiu and Bouché [1], and suggest the existence of six new cryptic *Postandrilus* species. As indicated before for other Lumbricidae genera [26], [27], *Postandrilus* seems to also need extensive systematic revision. Future work aiming to redefine this genus and list its species should rely on estimated phylogenetic relationships such as those presented here.

This study has revealed unprecedented earthworm species diversity in Majorca, considering the small size of the island (3,625 km^2^). Previous studies had already highlighted a remarkable earthworm diversity in other genera from the same (Lumbricidae: *Eisenia* [15], *Aporrectodea* [26], [28], *Allolobophora* [28], *Lumbricus* [28]) and different families (Megascolecidae: *Metaphire* [18], [29]; Hormogastridae: *Hormogaster* [16]), but the regions sampled in those studies were larger. Surprisingly, despite the relative small size of Majorca, *Postandrilus* seems to show a very patchy distribution since it was only found in 14 locations out of ∼30 sampled. Such distributional pattern may have accentuated isolation and subsequent allopatric and/or ecological speciation among populations leading to high number of cryptic lineages as revealed here and indicated before for other earthworms [16], [26], [28].

### Phylogeography of *Postandrilus*

*Postandrilus* species are only found in the Balearic Island of Majorca (western Mediterranean) and Galicia (NW Spain). This disjunct distribution could be the result of a vicariant event occurred in the Late Oligocene (30–28 Mya) that separated the Baleares-Kabylies microplate from the proto-Iberian Peninsula, or the result of a process of colonization of the Baleares from eastern Spanish *Postandrilus* populations during the Messinian salinity crisis (5.96–5.33 Mya), or a combination of both. Our phylogenetic trees did not show two deep subclades of Majorcan *Postandrilus* taxa, as expected under a two-step process of vicariance and subsequent dispersal. Instead all the insular *Postandrilus* formed a monophylum of umbellate shape, hence the vicariant-dispersal hypothesis can be rejected. Our molecular estimates indicate that the split between Majorcan *Postandrilus* and the related Iberian clade took place 30.1 Mya. This estimate agrees very well with the geological age estimated for the fragmentation of Baleares from the proto-Iberian Peninsula (vicariant hypotheses). Such time concordance would not be expected if *Postandrilus* evolved in situ in proto-Iberia and then colonized the Baleares (dispersal hypothesis). Our tree and time estimates also indicate that the proto-Iberian ancestor subsequently speciated into *P. bertae* and *A. morenoe* 22.6 Mya, while the Majorcan *Postandrilus* experienced a period of apparent evolutionary stasis, followed by a period of cladogenesis starting 5.7 Mya where many new lineages radiated simultaneously. This rapid diversification of the Majorcan *Postandrilus* matched the onset of the MSC (5.96–5.33 Mya), which apparently supports the dispersal hypothesis. We, however, believe that this estimate actually reflects the impact of the MSC on an already established insular lineage. The decrease of the sea level during the MSC could open new terrestrial habitats in Majorca. Then the subsequent reconnection of Mediterranean and Atlantic basins and rapid refill (a few years) of the Mediterranean [7] could contribute to the isolation of earthworm populations previously expanded and so, to their differentiation, as indicated above. A similar scenario could also be imagined under the dispersal hypotheses, but if we assume so, that would lead us to accept that the perfect concordance between the molecular and geological Oligocene estimates above are random. Additional biological and ecological evidence make also the dispersal hypothesis less plausible. Earthworms are presumed to have a dispersal ability of about 2–4 m/year [16] and *Postandrilus* is considered a sedentary and endogeic species. Mallorca is separated from Spain by ∼300 km, therefore earthworms would have to migrate during the entire duration of the MSC (∼63,000 years) at a rate of 4.8 m/year to reach the islands. Moreover, considering the salty origin of the land bridge connecting both landmasses, it is well possible that the soil conditions required for *Postandrilus* to survive and actively migrate to the Baleares were not given. There is some evidence of earthworms being transported by other animals [12]. They have, for example, been introduced to Dutch polders and New Zealand islands by birds [30], [31]. We have no evidence for (or against) it in the Baleares, but considering the subterranean lifestyle of *Postandrilus*, animal transportation seems also unlikely.

Further evidence of the vicariant origin of *Postandrilus* would first require finding new specimens in the Kabylies (Algeria). If the vicariant hypothesis were to be correct, as we expect, Kabylian and Majorcan *Postandrilus* should form a sister clade at least as old as the split between the Kabylies and Baleares microplates (∼21 Mya). Such sister relationship would also break the *apparent* initial evolutionary stasis of the Majorcan lineage. Furthermore, new *Postandrilus*–like specimens should be found in central and southeastern Spain more closely related to *P. bertae* and *A. morenoe* than to the Majorcan *Postandrilus*. Our current sampling efforts are focused on those two Spanish regions.

Finally, our results have also important implications for future earthworm phylogeographic and systematic studies. Earthworms have no hard body parts, hence they have barely left any useful fossil evidence in the paleontological record to calibrate the rate of evolution of their genes or time their radiation. Geological information is another alternative for calibrating molecular trees, but useful examples integrating both phylogenies and geology are scarce. This study provides calibrations for the rate of evolution of several commonly used mtDNA and nDNA genes in earthworms and confirms two geological calibrations for *Postandrilus*. Such information could be then used to time the origin and radiation of other lumbricids.

## Methods

### Ethics Statement

Majorcan earthworms were collected under a permit issued by the “Govern de les Illes Balears”, ref ALT69-71/2010. No specific permit was required to collect the Galician (NW Spain) specimens because the two sampled locations are not privately-owned or protected in any way and our field studies did not involve endangered or protected species.

### Earthworm Sampling

Forty-six specimens of *Postandrilus* representing all of the six described species and including 3–11 individuals per putative species were collected in 14 locations from Majorca and two from NW Spain (Table 1). These locations included six of the eight type localities in Majorca sampled by Qiu and Bouché [1]. We also intensively searched for *Postandrilus* in the Balearic Islands of Menorca and Ibiza, but no specimens were found.

**Table 1:** Taxon sampling, specimen codes, locality and coordinates.

Taxon	Code	Locality	Coordinates	
*Postandrilus lavellei*	PLAV434, 453, 454	Spain (S'Arenal, Majorca) – QB	N 39° 29′ 58.8″	E 2° 46′ 4.5″
*Postandrilus lavellei*	PLAV455–457	Spain (Ses Salines, Majorca) – QB	N 39° 21′ 32.8″	E 3° 02′ 30.6″
*Postandrilus lavellei*	PLAV458–460	Spain (Ses Salines, Majorca) – QB	N 39° 21′ 43.5″	E 3° 02′ 11.0″
*Postandrilus majorcanus*	PMAJ363	Spain (Puig de Maria, Pollença, Majorca)	N 39° 52′ 04.4″	E 3° 01′ 06.3″
*Postandrilus majorcanus*	PMAJ377	Spain (Musclo de ses Cordes, Majorca)	N 39° 53′ 47.0″	E 2° 55′ 8.13″
*Postandrilus majorcanus*	PMAJ378–379	Spain (Bosquet de Bóquer, Majorca) – QB	N 39° 54′ 44.6″	E 3° 05′ 23.2″
*Postandrilus majorcanus*	PMAJ386	Spain (Puig de Maria, Pollença, Majorca)	N 39° 52′ 04.4″	E 3° 01′ 06.3″
*Postandrilus majorcanus*	PMAJ448–472	Spain (Cala de Sant Vicenç, Majorca)	N 39° 55′ 02.0″	E 3° 03′ 15.1″
*Postandrilus majorcanus*	PMAJ449	Spain (Ariant, Majorca)	N 39° 54′ 13.9″	E 2° 57′ 20.0″
*Postandrilus medoakus*	PMED444, 450, 468	Spain (Mirador de ses Barques, Sóller, Majorca)	N 39° 47′ 26.3″	E 2° 43′ 31.7″
*Postandrilus palmensis*	PPAL466	Spain (Cap Salines, Majorca)	N 39° 16′ 35.6″	E 3° 03′ 32.0″
*Postandrilus palmensis*	PPAL383–385	Spain (Portocolom, Majorca) – QB	N 39° 27′ 00.0″	E 3° 14′ 00.0″
*Postandrilus palmensis*	PPAL360	Spain (Colonia de St Pere, Majorca)	N 39° 43′ 22.9″	E 3°18′ 32.9″
*Postandrilus palmensis*	PPAL374–376	Spain (Artá, Majorca) – QB	N 39° 41′ 00.9″	E 3° 21′ 00.5″
*Postandrilus palmensis*	PPAL469, 470, 474	Spain (Mirador de ses Barques, Sóller, Majorca)	N 39° 47′ 26.3″	E 2° 43′ 31.7″
*Postandrilus palmensis-lavellei*	PPA-LLAV446	Spain (Bosc de Bellver, Majorca)	N 39° 33′ 44.9″	E 2° 37′ 10.2″
*Postandrilus sapkarevi*	PSAP380–382, 477	Spain (Portocolom, Majorca) – QB	N 39° 27′ 00.0″	E 3° 14′ 00.0″
*Postandrilus sapkarevi*	PSAP475, 476	Spain (Caimari, Majorca)	N 39° 47′ 06.2″	E 2° 53′ 43.4″
*Postandrilus bertae*	PBER354, 482, 486, 488, 508	Spain (Pintos, Pontevedra)	N 42° 24′ 2.4″	W 8° 35′ 41.8″
*Postandrilus bertae*	PBER491, 492, 494	Spain (Cristo Rey, Pontevedra)	N 42° 23′ 13.4″	W 8° 34′ 24.4″
*Aporrectodea morenoe*	AMOR348	Spain (Sobradelo, Ourense)	N 42° 20′ 40.1″	W 6° 48′ 22″
*Aporrectodea morenoe*	AMOR503–505, 507	Spain (Covalos, Lugo)	N 42° 23′ 16.4″	W 7° 12′ 29.2″
*Cataladrilus edwardsi*	CEDW314	Spain (Castellfollit de la Roca)	N 42° 13′ 20.9″	E 2° 32′ 58.3″
*Cataladrilus monticola*	CEDW315	Andorra (Sant Julia)	N 42° 29′ 8.9″	E 1° 29′ 37.0″
*Prosellodrilus biauriculatus*	PRBIA321	France (Ariège)	N 42° 59′ 38.4″	E 1° 15′ 23.3″
*Prosellodrilus biseralis*	PRBIS323	France (Languedoc-Rousillon)	N 44° 4′ 18.7″	E 4° 47′ 15.6″
*Prosellodrilus pirenaicus*	PRPIR320	France (Ariège)	N 42° 59′ 27.9″	E 1° 13′ 26.1″

Type localities in Qiu and Bouché [1] are indicated by QB.

Until now, no one had studied *Postandrilus* evolutionary relationships, hence their closest relatives are unknown. Unpublished phylogenetic analyses performed by our group (available from the authors upon request) including *P. bertae*, *P. sapkarevi* and *P. majorcanus* (one specimen each), another 28 Lumbricidae genera, five non-lumbricid families (outgroups) and the same gene partitions used here plus 18S (∼800 bp) showed a strongly supported clade [bootstrap proportions (bp) = 100% and posterior probabilities (p*P*) = 1.0] clustering the three *Postandrilus, Aporrectodea morenoe*, three *Prosellodrilus* and two *Cataladrilus* species listed in Table 1. All of the other 13 *Aporrectodea* species and two *Cernovistovia* species included in the analyses fell in different clades. Hence, based on this up to date phylogenetic analyses of Lumbricidae evolutionary relationships, we have chosen here the *A. morenoe* and three *Prosellodrilus* and two *Cataladrilus* species listed in Table 1 as the outgroup.

### DNA Sequencing

Total genomic DNA was extracted using the DNAeasy Tissue kit (Qiagen). Regions of the nuclear 28S rDNA and mitochondrial 16S rDNA, 12S rDNA, NADH dehydrogenase (ND1), cytochrome oxidase subunit II (COII) and tRNA Asn, Asp, Val, Leu, Ala, Ser, and Leu genes were amplified using the polymerase chain reaction (PCR) and conditions in Pérez-Losada et al. [26]. PCR products were resolved by 1.5% agarose gel electrophoresis, visualized by SYBR Green, and purified using a MultiScreen PCRµ96 (Millipore) kit. Automated sequences were generated in both directions from different runs on an Applied Biosystems (ABI) 377XL automated sequencer. We used the ABI Big-dye Ready-Reaction kit and followed the standard cycle sequencing protocol, but using a 16th of the suggested reaction size. DNA sequences were deposited in GenBank under the Accession Numbers JN871915 – JN872139.

### Data Analysis

Nucleotide sequences from each gene region (all tRNAs were combined into a single gene region) were aligned using MAFFT v6 [32], [33] under the global (G-INS-i) algorithm and default settings. Phylogenetic congruence among gene regions (COII: 686 bp, 12S: 362 bp, 16S: 1200 bp, ND1: 917 bp, tRNAs: 402 bp, and 28 S: 809 bp) was assessed using the Wiens’ [34] protocol. No areas of strongly supported incongruence were observed among gene trees. Gene regions were then combined into four partitions: coding (COII and ND1), rRNA (12 S and 16 S), tRNAs and 28S. ML analysis of the concatenated partitions was performed in RAxML v7.2.0 [35] using 1,000 searches. JModelTest v1.0.1 [36] was used to select the appropriate models of evolution for each gene partition under the Akaike Information Criterion AIC [37]. The general time reversible model of evolution [38], with proportion of invariable sites and gamma distribution was selected for each data partition (GTR+Γ+I). Clade support was assessed using the non-parametric bootstrap procedure [39] with 5,000 bootstrap replicates run in the portal CIPRES Science Gateway portal [40].

The concatenated partitions were also analyzed using Bayesian methods coupled with Markov chain Monte Carlo (BMCMC) inference as implemented in MrBayes v3.1.2 [41]. Three independent BMCMC analyses were run in CIPRES with each consisting of four chains. Each Markov chain was started from a random tree and run for 10^7^ cycles, sampling every 1,000^th^ generation. Model parameters were unlinked and treated as unknown variables with uniform default priors and they were estimated as part of the analysis. Convergence and mixing were monitored using Tracer v1.5 [42]. All sample points prior to reaching stationary were discarded as burn-in. The posterior probabilities for individual clades obtained from separate analyses were compared for congruence and then combined and summarized on a 50% majority-rule consensus tree.

Divergence times for the clades of interest were estimated in BEAST v1.6.1 [43]. We used the four partitions described above. The GTR+Γ+I model of nucleotide substitution and the relaxed lognormal model of rate of substitution [44] were chosen for each data partition. A Yule speciation prior was used for the tree prior as recommended by the authors. No fossils exist to calibrate the *Postandrilus* radiation, hence instead we used species divergence estimates based on geological events to define priors for the rates of substitution in each partition. Recently, Novo et al. [17] estimated the substitution rates of the 16S-tRNA and 28S genes in Hormogastridae earthworms from the eastern Iberian Peninsula and Corsica and Sardinia. They assumed that the cladogenic event leading to the split between the Iberian and insular species was due to the separation of the CSCP microplate from proto-Iberia, the same cladogenic event we are trying to date here using *Postandrilus* from Baleares. Hence, to avoid the circularity of using Novo et al. [17] rate estimates, we used an independent calibration based on the work of Chang and Chen [45] and Chang et al. [18] for *Metaphire* (Oligochaeta, Megascolecidae) from Taiwan. Using their COI and ND1 sequences and calibration, we re-estimated the rates for these two genes using BEAST, which we then combined into a normal distribution of mean 2.5% substitutions(s)/My^-1^ and SD = 0.6% s/My^−1^ and used for our coding partition (COII and ND1 combined). Previous studies have shown similar levels of genetic divergence for COII and COI in lumbricids (i.e., similar rates of substitution) [15], [26], [46]. No rates have been suggested for ribosomal or transfer RNA genes in earthworms (except [17]), however it has been noticed that these genes do not evolve as fast as mtDNA coding genes [15], [26], [29]. We then used a similar approach to that described by J. Thorne in the software multidivtime for estimating rates of molecular evolution and divergence times. First, we estimated all the root-to-tip ML tree lengths for each non-coding gene in TreeStat v1.6.1 (part of the BEAST package) and then used the median of those lengths to generate normal prior distributions for each partition. The resulting normal priors [mean (SD) %] relative to the coding partition prior were as follows: rRNA = 1.2 (0.3) s/My^−1^, tRNA = 0.7 (0.16) s/My^−1^, and 28S = 0.06 (0.015) s/My^−1^. These rates and the rate for the coding partition above agree well with those estimated in other invertebrates including annelids [19], [20]. Two runs 2x10^7^ generations long were completed and combined using LogCombiner v1.6.1 (part of the BEAST package). All the output generated by BEAST was analyzed in Tracer v1.5.

Confidence in our best hypotheses of phylogenetic relationships were tested by first creating alternative hypotheses (e.g., *Postandrilus* is monophyletic; see below) in MacClade as indicated in Pérez-Losada et al. [47] and then comparing them under both likelihood and Bayesian frameworks. Likelihood topological tests were conducted using the Shimodaira and Hasegawa (S–H) [48] test as implemented in RAxML. Bayesian topological tests were performed as described in Huelsenbeck et al. [49].

Several methods for empirically testing species boundaries have been proposed and compared [50], [51], [52], [53], [54]. Here we used an integrative approach of species delimitation that takes into account multiple lines of evidence by combining phylogenetic relatedness with other factors like shared morphological and ecological evidence. This general integrative approach has been reviewed and argued for and explicitly applied by several researchers [23], [24], [25], [26].
